# Accurate Determination of Moisture Content in Flavor Microcapsules Using Headspace Gas Chromatography

**DOI:** 10.3390/polym14153002

**Published:** 2022-07-25

**Authors:** Xueyan Liu, Chuxing Zhu, Kang Yu, Wei Li, Yingchun Luo, Yi Dai, Hao Wang

**Affiliations:** 1 School of Chemical Engineering, Guizhou Minzu University, Guiyang 550025, China; liu1996@gzmu.edu.cn (X.L.); yukang@gzmu.edu.cn (K.Y.); liwei1998@gzmu.edu.cn (W.L.); 05111583@gzmu.edu.cn (Y.L.); 2 State Key Laboratory of Pulp and Paper Engineering, South China University of Technology, Guangzhou 510641, China; 202010105766@mail.scut.edu.cn; 3 Technology Center, China Tobacco Yunnan Industrial Co., Ltd., Kunming 650231, China

**Keywords:** tracer water, flavor microcapsule, headspace, gas chromatography

## Abstract

This study demonstrates an accurate method for determining the moisture content in flavor microcapsules using headspace gas chromatography. The method involves measuring the gas chromatography signals of water from vapor in a headspace vial containing flavor microcapsules at a temperature of 125 °C. The measurements were recorded over four headspace extractions, from which the moisture content in the microcapsule samples was extrapolated via simple vapor-phase calibration. The results revealed that the proposed method demonstrated good precision (a relative standard deviation of <3.11%) and accuracy. The proposed method is accurate, highly sensitive, automated, and suitable for testing the moisture content of flavor microcapsules and related products.

## 1. Introduction

Microcapsules, with sizes ranging between 1 and 1000 μm, are composed of a core (internal part) and shell (external part) [1,2]. The physical properties of the microcapsule shell effectively protect and control the release of sensitive core materials under a variety of conditions. Therefore, they have wide applications in several fields, such as medicine, agriculture, chemical industries, pharmaceuticals, and food engineering [3,4,5,6,7,8]. To prepare microcapsules, the materials and processing conditions are important for product quality [9]. Additionally, the amount of water in microcapsules, regarded as an impurity, is critical to their quality and performance [10]. For example, if the content of water in flavor microcapsules is extremely high, the viscosity of the shell materials increases, affecting storage and flavor release in related products [11,12,13]. Therefore, a method that can effectively determine the moisture content of flavor microcapsules is crucial for their production and quality control.

Conventionally, the water content in flavor microcapsules is determined using the direct oven-drying method [14], in which the water in the flavor microcapsule is removed through evaporation at approximately 105 °C for 3 h in an oven. The amount of water lost can be obtained by weighing the sample before and after drying, thereby allowing the calculation of the moisture content in the sample. However, because certain volatile compounds, such as ethyl acetate and ethanol, are also present in microcapsules, they may be removed during the drying process and can cause significant errors in water content measurements [15]. This issue can be addressed using the Karl Fischer titration method [16], in which a reaction occurs between the titration reagent (SO_2_ or I_2_ in CH_3_OH and pyridine or imidazole buffer medium) and water in the sample. This method demonstrates better reproducibility and is sensitive to the moisture content in the sample compared to the oven-drying method. However, in addition to the requirements of a complex titrator, expensive reagents, and time-consuming procedures complicating this method, certain compounds containing carbonyl groups (commonly present in flavorings) in the matrices can react with the Karl Fisher reagent, affecting measurement accuracy [17]. The low-temperature dry nitrogen purge method has also been applied to determine the water content of microcapsules [18]. In this method, a flavor microcapsule sample was placed in a dish in a test chamber (d = 35 cm, H = 6 cm) maintained under a controlled atmosphere (temperature = 40 °C, relative humidity = 1%), and the water in the sample was eliminated by injecting heated absolute dry nitrogen. By recording the weight at each hour, until the ratio of the initial mass change is less than 0.04%, the water content of the sample can be calculated. This method is accurate; however, it takes longer than 16 h to test one sample, which significantly affects its detection efficiency. Gas chromatography (GC) with a thermal conductivity detector (TCD) was also proposed for determining the water content of microcapsule samples [19]. Herein, the water peak was well separated via GC; therefore, the water content could be quantified. However, before GC measurements can be performed, complicated and time-consuming sample pretreatment procedures, such as solvent extraction and separation, are mandatory. This involves the extraction of the sample with isopropanol and filtering of the supernatant liquid using an organic phase filter membrane before quantification using GC.

Headspace gas chromatography (HS-GC), which is different from the conventional GC technique, is an effective tool to detect volatile analytes in solid or liquid complex matrices [20,21]. When equipped with a TCD, HS-GC can be used to determine the water content of samples. However, traditional HS-GC analysis cannot be used to determine the water content in solid samples because the vapor–solid partitioning (K) of water varies with different sample matrices, which introduces errors in the method calibration. Multiple headspace extraction (MHE) modes are available in several commercial headspace auto-samplers [22,23,24]. For each headspace extraction, the analyte in the vapor phase was partially removed from the vial for GC analysis. If the analyte in the matrices is almost completely vaporized in the vial, the total amount of analyte can be calculated by integrating the contents during the MHE. Consequently, the sample–matrix effect can be eliminated. This suggests that the MHE-GC technique can be used to determine the moisture content of flavor microcapsules.

The objective of this study was to develop an accurate method for the determination of moisture content in flavor microcapsule samples using MHE-GC. The major focus areas involved the establishment of a methodology and determination of the effects of GC conditions, equilibration time, temperature, sample size, and extraction number on the measurement of moisture content. To evaluate the accuracy of this method, microcapsule samples with different flavors were analyzed using MHE-GC, and the results were compared with those obtained using reference methods.

## 2. Experimental Section

### 2.1. Materials

Flavor microcapsule samples were collected from EnDian Science and Technology Development of Yunnan Co., Ltd. (Kunming, China). The polymer shell materials were prepared using carboxymethyl chitosan and sodium alginate. The core materials were composed of flavor components and octyl and decyl glycerates. Distilled water was obtained from a laboratory device. All flavor microcapsule samples were stored in sealed bags prior to the analysis.

### 2.2. Equipment and Operation Procedures

The HS-GC measurements were performed using an automated headspace sampler (DANI HS 86.50 PLUS, Cologno Monzese, Italy) and GC system (Agilent GC 8860A, Santa Clara, CA, USA) equipped with a TCD and capillary column (Model GS-Q, 30 m length × 0.3 μm i.d, 530 μm thickness), J&W Scientific, Folsom, CA, USA). The volumes of the headspace vials and sample loop were 21.6 and 3 mL, respectively. The MHE-GC measurement conditions were as follows: the temperatures of the oven, sample loop, and transportation line were 125, 130, and 135 °C, respectively; both the injector and detector temperatures were 250 °C; the GC injection mode was split, and the split ratio was 10:1; the carrier gas was nitrogen with a flow rate of 25 mL/min; the vial pressurization time was 0.2 min; the loop equilibration time was 0.05 min, and the sample-loop time was 0.2 min; the carrier gas pressure and pressurization were 1.5 and 2.00 bar, respectively; the headspace vial was strongly shaken during equilibration.

### 2.3. Measurement Procedures

**Figure 1:**
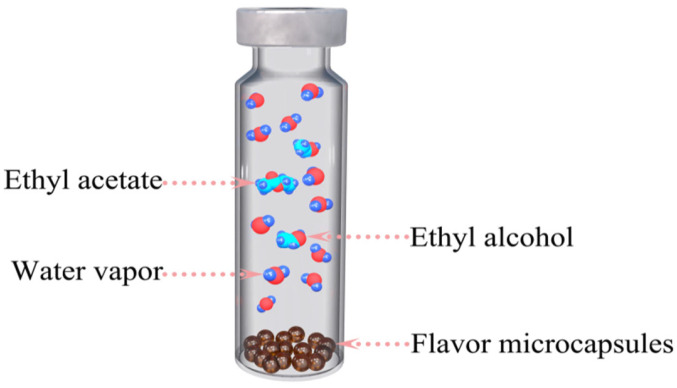
Schematic of the headspace vial used to measure the moisture content in microcapsule samples.

Approximately 0.20 g of the microcapsule sample was added to a dried and empty headspace vial, as presented in Figure 1. The weight of the microcapsule sample was determined by weighing the sample vial (including the PTFE/silicone septum and aluminum cap) before and after sample addition. Subsequently, the sample vial was immediately sealed and transferred to a headspace autosampler for MHE-GC testing. The sample vials were equilibrated at 125 °C, and the interval time for MHE-GC measurements was 6 min. Accordingly, the GC signals of the water vapor (peak area) were measured using GC-TCD for each headspace extraction.

### 2.4. Determination of Moisture Content Using the Low Temperature Dry Nitrogen Purge Method

The water content in the microcapsule sample was determined using the low-temperature dry nitrogen purge method. The procedure was as follows: the temperature and relative humidity in the chamber were (40 ± 0.1) °C and below 1.0%, respectively. Approximately 5 g of the sample was added to a dish, and the water in the sample was eliminated by injecting heated absolute dry nitrogen. The weight was recorded every hour until the ratio of the mass change to the initial sample mass was less than 0.04%. The water content in the microcapsules (*R*) was calculated using the following Equation (1):

(1) $$R=\frac{m_{0}-m_{1}}{m_{0}}\times 100\%$$

where *m*_0_ denotes the weight of the microcapsule sample added to the dish, and *m*_1_ denotes the weight of the sample after reaching a constant weight.

### 2.5. Determination of Moisture Content Using the Traditional Oven Drying Method

The moisture content of the microcapsule samples was also determined using the oven-drying method. In this method, the test was conducted as follows: approximately 5 g of the sample was added to a dish and placed in a drying oven at 105 °C for approximately 4 h. The dish was then placed in a dryer and allowed to cool to room temperature. This procedure was repeated until a constant weight was obtained. The moisture content in the microcapsules (*R*) was calculated using the following Equation (2):

(2) $$R=\frac{m_{0}-m_{1}+m_{2}}{m_{0}}\times 100\%$$

where *m*_0_, *m*_1_, and *m*_2_ denote the weights of the microcapsule sample, dish after reaching a constant weight, and the total weight of the dish and microcapsule sample after reaching a constant weight, respectively.

## 3. Results and Discussion

### 3.1. Theory of the MHE-GC Method

**Figure 2:**
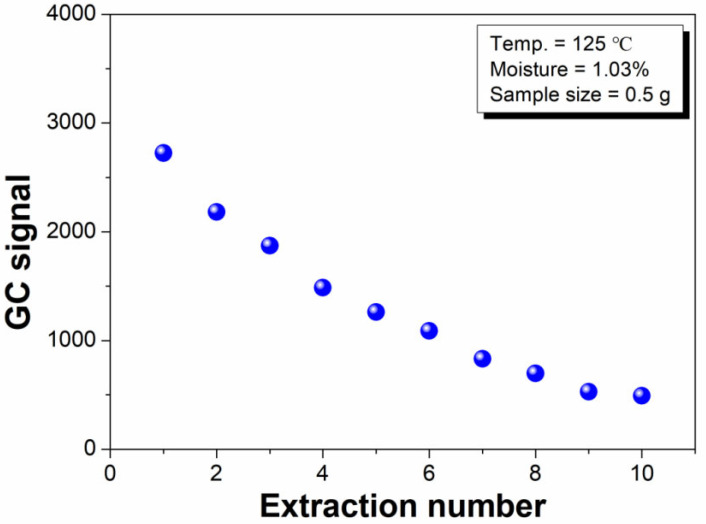
Relationship between the GC signal of water and extraction number.

When a given amount of the flavor microcapsule sample in the sealed headspace vial reached phase equilibrium at an elevated temperature (>100 °C), water co-existed in the solid and gas phases. Using the MHE-GC technique, consecutive analyses were conducted on the same sample vial, from which a part of the water in the gas phase could be extracted. As illustrated in Figure 2, the GC signal intensity of water gradually decreases with more extractions [25,26,27], which indicates that the entire water content in the microcapsule sample can be completely withdrawn if the extraction number (n) is sufficiently high.

According to MHE-GC theory [27], the relationship between *A_n_* and *n* can be described as Equation (3):

(3) $$Log\left(A_{n}\right)=Log\left(A_{0}\right)-b_{n}$$

where *A_n_* and *A*_0_ denote the GC signals at *n* and 0 extractions, respectively, and *b* denotes the slope obtained from the linear fit. Therefore, the integrated GC signal (*A_t_*) can be expressed as Equation (4):

(4) $$A_{t}=A_{1}+A_{1}10^{-b}+\ldots +A_{1}10^{-b\left(n-1\right)}=\underset{n\to \infty }{\lim}\frac{A_{1}(1-10^{-bn})}{1-10^{-b}}=\frac{A_{1}}{1-10^{-b}}$$

where *A_t_* denotes the sum of the peak areas, and *A*_1_ indicates the GC signal of the first extraction.

The total mass of water in the gas phase (*m_t_*) is linearly proportional to *A_t_* in Equation (5):

(5) $$m_{t}=KA_{t}$$

where *K* can be obtained using the calibration method.

Therefore, based on Equations (4) and (5), the tracer water content in the microcapsule sample can be calculated as Equation (6):

(6) $$R=\frac{KA_{1}}{W(1-10^{-b})}\times 100\%$$

where *R* denotes the content of tracer water in the microcapsule sample, and *W* denotes the weight of the sample added to the headspace vial.

### 3.2. Selection of HS-GC Measurement Conditions

#### 3.2.1. Conditions for GC Separation

**Figure 3:**
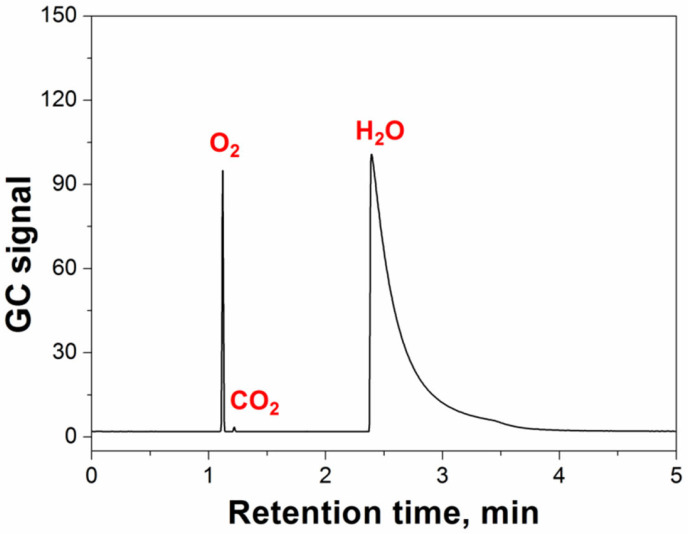
Chromatogram of a microcapsule sample.

Effective GC separation for water and other volatile substances is required to determine the water content of the microcapsule samples. Figure 3 shows the chromatogram of a microcapsule sample. The water and oxygen (in air) peaks could be separated well under the given GC operation conditions, and no other volatile organic compounds were observed in the chromatogram. These results further support that the GC-TCD system can effectively prevent interference resulting from complex volatile organic compounds in the matrix while determining the amount of water in the samples.

#### 3.2.2. Temperature Conditions and Interval Times in Headspace Equilibration

**Figure 4:**
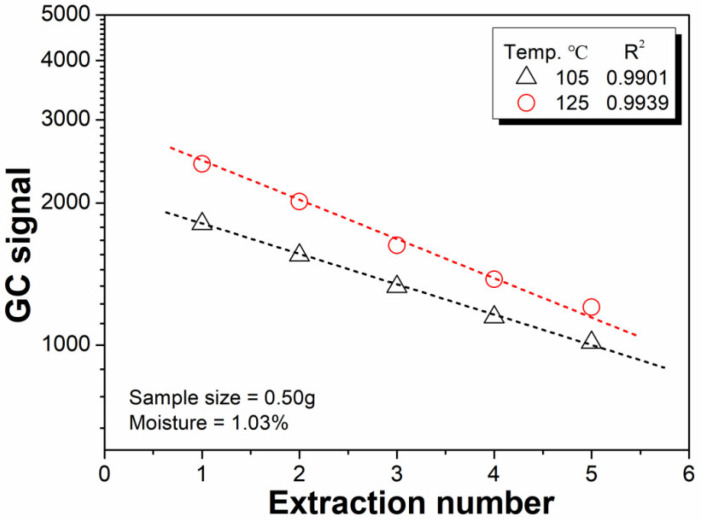
Effect of equilibrium temperature on the water removal.

The MHE is based on the gradual removal of water from microcapsules. Therefore, the equilibrium temperature should be higher than the boiling point of water (>100 °C). However, if an extremely high equilibration temperature is applied during the test, it may lead to a high pressure in the sealed headspace vial and increase the risk of leakage [28,29]. Therefore, the feasible equilibration temperature was deemed to be less than 130 °C. Figure 4 depicts the effect of equilibrium temperature on the water lost from a given microcapsule sample during the MHE process, in which two temperatures, 105 °C and 125 °C, were selected. The high temperature (125 °C) accelerated the water removal rate during the sample shaking process, as revealed in other studies [22,25]. Therefore, 125 °C was selected as the equilibration temperature for the subsequent study.

**Figure 5:**
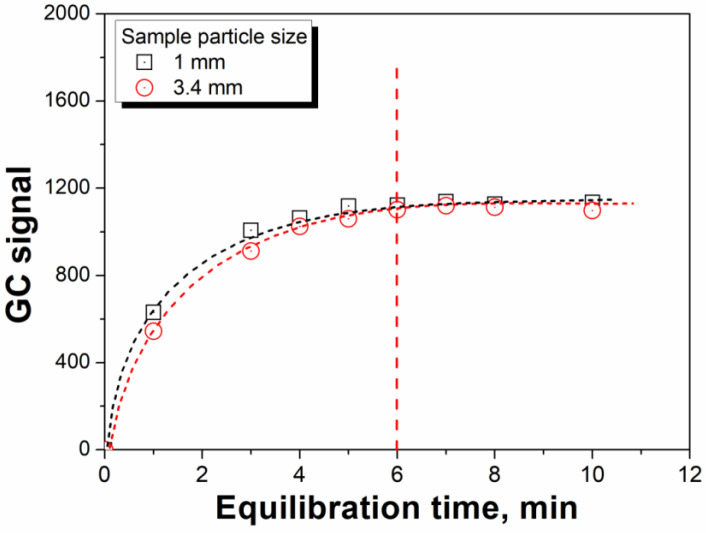
Time required to reach the water headspace equilibrium.

Because the present method is based on the equilibrium of water partitioning between the vapor and solid phases, the time required for equilibration before each headspace extraction must be determined. Figure 5 shows the GC signals of water detected at different time intervals in the headspace vial at a temperature of 125 °C. Equilibrium was attained in 6 min for the microcapsule samples, in which two sample particle sizes of 1 and 3.4 mm were selected. Notably, this equilibrium time is longer than that required for the determination of moisture content in paper materials [22]. This may be related to the structure of the microcapsule shell, which effectively protects the tracer water from the microcapsule release. Therefore, 6 min was chosen as the time interval for the MHE process.

#### 3.2.3. Selection of the Sample Size for Headspace Measurements

**Figure 6:**
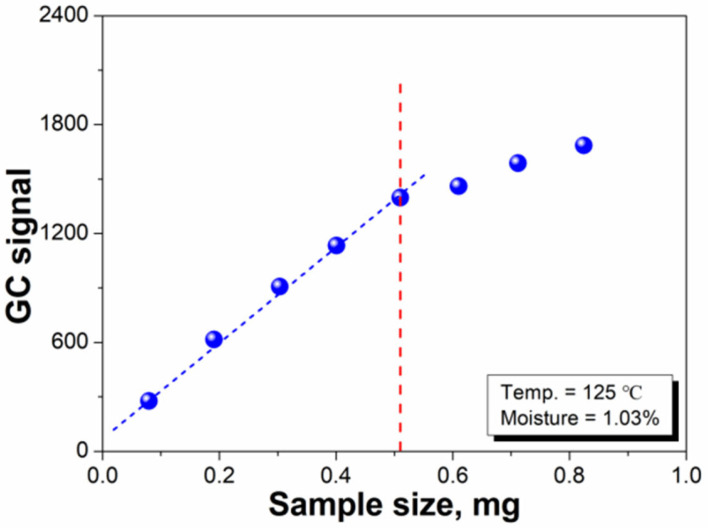
Effect of the sample size on the water GC signal.

In MHE-GC measurements, a large sample weight is helpful for improving the sensitivity of the method [30]. However, using a larger sample size (weight) can cause lower detection efficiency because of the slower release of water from the core material in the flavor microcapsule sample [22]. As mentioned in Section 3.1, the value of *A*_1_ is crucial for calculating the water content in the microcapsule samples. Figure 6 illustrates the effect of the sample size on the water GC signal. A linear variation in the GC signal with respect to the sample size was observed from approximately 0 to 0.51 g. When the sample amount was more than 0.51 g, the *A*_1_ value was lower than the actual value, resulting in an error in the test results. This outcome is because the total gas pressure in the headspace bottle is very high, resulting in pressure effects [30,31]. Therefore, 0.51 g was determined to be the maximum sample content for successfully measuring the moisture content in the samples.

#### 3.2.4. Selection of the Extraction Number in the MHE Process

**Figure 7:**
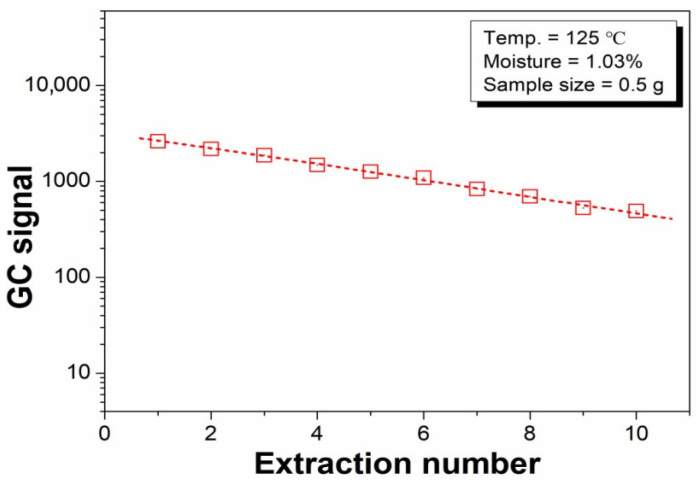
Relationship between the logarithm of the water GC signal and extraction number.

As mentioned in Section 3.1, the value of b in Equation (4) is important for calculating the water content of the microcapsule samples. Figure 7 illustrates the logarithm of the water GC signal versus the extraction number. Consistent with previous studies [32], we obtained a linear relationship between the logarithm of the water GC signal and extraction number. The b value from the linear fit of the 1st to 10th data points is acceptable. The accuracy of the present method can be improved by increasing the extraction number; however, a greater number of extractions leads to longer measurement times, which results in a lower detection efficiency and an increased risk of air leakage. As a compromise, four headspace extractions were selected in the present study.

### 3.3. Method Evaluation

#### 3.3.1. Method Calibration 

The HS-GC method was calibrated using a vapor phase calibration technique [22]. Calibration was conducted by adding 0–10.0 mg of distilled water into a set of headspace vials. These vials were tested using the MHE-GC method. Subsequently, the standard external vapor calibration relationship was obtained as follows:

(7) $$A=190\left(\pm 2.27\right)+2003\left(\pm 9.01\right)\times m\;(n=7,R^{2}=0.9989)$$

In other words, *A* = *a*(Δ*a*) + *s*(Δ*s*) × *m*, where *A* represents the integrated GC peak areas of water in the MHE-GC measurements, and *m* denotes the mass (mg) of distilled water added to the headspace vial. *s*, *a*, and Δ*a* represent the slope, intercept, and uncertainty, respectively, of the intercept of Equation (7).

The limit of quantitation (*LOQ*) of the method was 0.0461 mg, which was calculated using Equation (8) [33]. If 0.2 g of microcapsules were used in the testing, the *LOQ* was approximately 0.0231%. Clearly, the MHE-GC method is highly sensitive and meets the requirements for moisture content testing in the production of flavor microcapsules and related products.

(8) $$LOQ=\frac{a+10\left|\Delta \alpha \right|}{s}$$

#### 3.3.2. Reproducibility of the Measurements

The reproducibility of the MHE-GC method was investigated by testing three different microcapsule samples in triplicates. As presented in Table 1, the relative standard deviation (RSD) of the water content measured using the proposed method was less than 3.11%, which indicates that the method demonstrates good precision.

#### 3.3.3. Method Validation

To verify the performance of the present MHE-GC method, seven different particle sizes and types of flavor microcapsules were analyzed using the traditional oven-drying method [14], low-temperature dry nitrogen purge method [18], and MHE-GC method. As presented in Table 2, the results obtained using the low-temperature dry nitrogen purge method matched well with those obtained via the MHE-GC method (relative difference < 5.5%). Importantly, the difference in results is considered minor and significantly below the acceptable values of method errors (e.g., 15%), as revealed through other methods [34,35]. Meanwhile, the values obtained using the MHE-GC method are lower than those obtained through the traditional oven-drying method, which is likely caused by the mass loss of sensitive volatile compounds at high temperatures. Therefore, the traditional oven-drying method is not reliable for determining the moisture content of flavor microcapsules. Conversely, the MHE-GC method is accurate, efficient, and justifiable for the determination of water content in flavor microcapsule samples.

## 4. Conclusions

An accurate method for determining the moisture content of flavor microcapsule samples was introduced based on the multiple headspace extraction gas chromatography technique. Compared with the traditional oven drying method, the significant advantage of the MHE-GC method is that it is extremely accurate, as it can effectively eliminate the interference caused by the loss of volatile organic compounds in the sample at high temperatures. The results revealed that the proposed method has good precision (a relative standard deviation of <3.11%) and accuracy. The present method is simple and automated and can serve as a reliable tool for testing the moisture content of flavor microcapsule samples and related products.

## Figures and Tables

**Table 1:** Reproducibility of the MHE-GC method.

Replica No.	Water Content, %		
	Sample 1	Sample 2	Sample 3
1	1.50	1.13	1.74
2	1.57	1.2	1.71
3	1.56	1.15	1.68
RSD, %	2.45	3.11	1.75

**Table 2:** Comparison of methods.

Sample ID	Water Content, %			Relative Error, %	
	Low Temperature Dry Nitrogen Purge Method (n = 3)	Oven Drying Method (n = 3)	MHE-GC (n = 3)	Low Temperature Dry Nitrogen Purge Method	Oven Drying Method
1	0.86 ± 0.01	1.01 ± 0.02	0.87 ± 0.02	1.16	−13.9
2	1.12 ± 0.01	1.32 ± 0.01	1.08 ± 0.01	−3.57	−18.2
3	1.40 ± 0.02	1.66 ± 0.03	1.35 ± 0.02	−3.57	−18.7
4	1.66 ± 0.02	1.93 ± 0.02	1.64 ± 0.02	−1.20	−15.0
5	1.99 ± 0.03	2.14 ± 0.03	2.06 ± 0.02	3.52	−3.7
6	1.09 ± 0.03	1.38 ± 0.02	1.03 ± 0.01	−5.50	−25.4
7	2.13 ± 0.01	2.36 ± 0.03	2.05 ± 0.01	−3.76	−13.1

## Data Availability

The raw/processed data required to reproduce these findings cannot be shared at this time, as the data also form part of an ongoing study.
